# Phenotype and Genetics of Spinocerebellar Ataxia Type 27B: Novel Movement-disorder Features, Cognitive Impairment, and Repeat Expansion Findings

**DOI:** 10.1007/s12311-026-02041-y

**Published:** 2026-06-29

**Authors:** Ronak Rashedi, Franca Peemöller, Hannes Erdmann, Mathias Gelderblom, Ute Hidding, Christos Ganos, Robert Chen, Angela Abicht, Simone Zittel

**Affiliations:** 1 Department of Neurology, University Medical Center Hamburg-Eppendorf, Martinistr. 52, Hamburg, 20246 Germany; 2 Medical Genetics Center (MGZ) Munich, Munich, 80335 Germany; 3 Friedrich Baur Institute, Department of Neurology, LMU University Hospital, Ludwig-Maximilians-Universität München, Munich, 80336 Germany; 4 Edmond J. Safra Program in Parkinson’s Disease, Division of Neurology, Movement Disorder Clinic, University of Toronto, Toronto Western Hospital, Toronto, ON Canada; 5 Division of Neurology, Department of Medicine, Krembil Research Institute, University of Toronto, University Health Network, Toronto, ON Canada

**Keywords:** Spinocerebellar Ataxia, Cerebellar Cognitive Affective Syndrome, Repeat Expansion Disorders

## Abstract

Spinocerebellar ataxia type 27B is a recently described autosomal dominant, late-onset cerebellar ataxia caused by an intronic GAA repeat expansion in the fibroblast growth factor 14 (*FGF14*) gene. Despite being recognized as a frequent adult-onset ataxia, its full clinical spectrum remains incompletely understood. To characterize the neurological, cognitive, and paraclinical phenotype of patients with heterozygous *FGF14* repeat expansions (>200) and expand the currently known motor and non-motor features, as well as to assess the co-occurrence of other repeat expansions. In this cross-sectional single-center study, patients with heterozygous *FGF14* repeat expansions underwent standardized neurological examination and cognitive screening. Paraclinical data were reviewed when available. 18 patients were included in the study (mean age at onset: 64 [37–79], at examination: 76 [61–94]). They all presented with gait ataxia, most commonly a lateral veering gait with corrective sidesteps. In addition to the core known cerebellar phenotype, we identified other movement-disorder manifestations, including myokymia, myoclonus, dystonia, and parkinsonism, with nigrostriatal degeneration confirmed in one patient. Cognitive impairment was common, with two-thirds of patients fulfilling criteria for cerebellar cognitive–affective syndrome (mean MoCA: 25 [21–29], CCAS: 86.9/120 [62–108]). Worse CCAS and MoCA performance was associated with increasing ataxia severity. *FGF14* repeat expansions ranged from 210 to 520, and co-occurrence with heterozygous expansions in *RFC1* or *ATXN8/ATXN8OS* were identified in three patients. Earlier diagnostic misclassification as transient ischemic attack was reported in 33%. These findings expand the known phenotype of spinocerebellar ataxia type 27B, emphasizing it as a multisystem movement disorder.

**Supplementary Information:**

The online version contains supplementary material available at 10.1007/s12311-026-02041-y.

## Introduction

Spinocerebellar ataxia type 27B (SCA27B), first reported in 2023, is an autosomal dominant, late-onset, slowly progressive cerebellar ataxia caused by an intronic GAA repeat expansion in the fibroblast growth factor 14 (*FGF14*) gene [1, 2]. The diagnosis is confirmed in symptomatic patients with a heterozygous (GAA)_>300_ repeat expansion. Repeat expansions of (GAA)_250−300_ are considered to have reduced penetrance and may establish a confirmed genetic diagnosis in patients with a compatible phenotype [3]. Nevertheless, recent studies suggested a lower pathological threshold of (GAA)_200−250_ repeat expansions for the disease [4].

Despite its recent description, SCA27B is now considered one of the most prevalent causes of late-onset cerebellar ataxias [5, 6]. Clinically, SCA27B typically manifests in the sixth or seventh decade of life and is characterized by gait and stance ataxia, dysarthria, and cerebellar oculomotor abnormalities, particularly downbeat nystagmus [7]. In contrast to many other hereditary cerebellar ataxias, SCA27B often presents with episodic symptoms or a fluctuating disease course, with exacerbations triggered by stress, caffeine, fatigue, or alcohol, before progressing to a persistent cerebellar syndrome [8, 9]. In some cases, postural tremor and peripheral neuropathy have also been described [10].

Despite the growing number of reported SCA27B cases, the phenotypic spectrum of this disorder is still being delineated. In particular, movement-disorder manifestations beyond the classic cerebellar syndrome appear to be underrecognized. Moreover, the cognitive profile of SCA27B has received little attention. Although the cerebellum is well recognized as a key structure in cognitive and affective regulation, data on cognitive impairment and cerebellar cognitive–affective syndrome (CCAS) in SCA27B remain sparse [11].

Hence, the present study aimed to provide a systematic clinical characterization of a cohort of patients with heterozygous repeat expansion in the *FGF14* gene, with a particular focus on expanding the known clinical phenotype. The objectives in this study were (i) to investigate the repeat expansion threshold compatible with SCA27B phenotype and the frequency of other repeat expansions, (ii) to describe previously unreported motor features, and (iii) to systematically assess cognitive involvement using the Montreal Cognitive Assessment (MoCA) and CCAS screening, thereby providing novel insights into the motor, cognitive and genetic spectrum of SCA27B.

## Methods

### Study Design

This was a cross-sectional study carried out at the Movement Disorders Outpatient Clinic of the Department of Neurology of the University Medical Center Hamburg-Eppendorf. We screened all patients with late-onset ataxia, defined by symptom onset > 40 years [12], who had been in regular clinical follow-up between January 2024 and December 2025. Patients with a heterozygous repeat expansion in the *FGF14* gene were then invited for a second systematic visit and standardized examination.

### Participants

Eighteen patients fulfilled the inclusion criteria, defined as:

a clinical diagnosis of late-onset cerebellar ataxia, and.
b confirmation of an *FGF14* (GAA) _>200_ repeat expansion.

Patients with alternative genetic, metabolic, structural, inflammatory, or immune-mediated causes of ataxia were excluded.

### Genetic Analysis

The genetic analysis was done using CRISPR/Cas9-targeted Oxford Nanopore Technology (ONT) long-read sequencing (Clin-CATS assay), as described previously [13, 14].

The genetic confirmation of SCA27B was based on the detection of a pure GAA-repeat expansion in *FGF14* exceeding the threshold of 300 repeat expansions. Patients with *FGF14* repeat expansions between 200 and 300 were assumed to be positive for SCA27B, when presented with a respective phenotype. Simultaneous repeat expansion in other genes was documented when applicable.

### Clinical Assessment

For each participant, a comprehensive neurological examination was performed by experienced movement-disorder neurologists (RR, SZ) following a pre-defined assessment protocol and questionnaire. Ataxia severity was quantified using the Scale for the Assessment and Rating of Ataxia (SARA) [15], and extracerebellar manifestations were documented using the Inventory of Non-Ataxia Signs (INAS) [16].

Cognitive function was assessed using the MoCA [17] and the CCAS screening [18]. Self-reported non-motor symptoms, episodic ataxia signs, and response to 4-aminopyridine were systematically documented during a structured interview. During this interview, demographic information, as well as the detailed medical history were also recorded.

Where available, results of paraclinical investigations, including brain MRI and neurophysiological investigations, were reviewed and documented.

### Ethical Considerations

This study was conducted in accordance with the Declaration of Helsinki and approved by the local ethics Committee of Hamburg (2025–101523-BO-ff). All participants provided written informed consent.

### Statistical Analysis

Clinical data are reported descriptively. Continuous variables are reported as means and ranges. No formal group comparisons were performed. When applicable, correlation analysis was performed using Spearman’s rank correlation coefficient, using SPSS Statistics, version 26. A p-value < 0.05 was considered statistically significant.

## Results

Of 99 patients identified, a total of 74 patients with late-onset ataxia were screened for SCA27B. Eighteen patients were identified as carriers of a heterozygous *FGF14* GAA median repeat expansion of more than 200, who were invited for the systematic interview and standardized examination. Two patients were identified with *FGF14* repeat expansions below the currently suggested pathological range, with 175–200 and 180–185 repeat expansions, respectively, and were therefore excluded from our study.

A comprehensive list of clinical diagnoses within the screened late-onset ataxia cohort is provided in Supplementary Material 1.

### Patient Characteristics

Eighteen patients with heterozygous repeat expansions in *FGF14* were included in the study (11 women, 7 men). The mean age at symptom onset was 64 years (range: 37–79), and the mean age at diagnosis was 76 years (range: 61–94). Age at diagnosis and age at examination were identical in all patients, as the genetic diagnosis was established in the same year of clinical assessment. Five patients (5/18) reported a positive family history of ataxia. Additionally, five (5/18) reported a family history of gait disturbance without an established diagnosis.

### Genetic Findings

The *FGF14* repeat lengths in our cohort ranged from 210 to 520 repeats. Eight patients had heterozygous GAA repeat expansions exceeding 300 repeats, consistent with a fully penetrant pathogenic expansion; seven had repeat expansion sizes within the reduced penetrance range of 250–300 repeats, and three had repeat expansion lengths within the range of 200–250 repeats. One patient with a disease onset at age 37 years carried pathogenic GAA repeat expansions of 320–360 repeats on the first allele of the *FGF14* gene, along with a smaller repeat expansion of 210–230 repeats on the second allele. Three individuals carried co-occurring heterozygous repeat expansions (*RFC1*: 345–365 AAGGG repeats; *ATXN8/ATXN8OS*: 63(+/−1) repeats, and 150–155 repeats). The genetic findings are presented in detail in Table 1.

**Table 1:** Demographic and Genetic Characteristics of the SCA27B patients

Patient No.	Sex	Age at Symptom Onset	Age at Diagnosis/Examination	Genetic findings	Family history for ataxia	Suspected Ischemic events in the past	Treatment with Fampridine (4-Aminopyridine)	Clinical response to Fampridine
1	F	61	74	Heterozygous, 290–310 GAA repeats			+	+
2	F	66	69	Heterozygous, 250–290 GAA repeats	+ (Father, Brother)		-	
3	F	60	61	Heterozygous, 280–300 GAA repeats	+ (Sister)		+	-
4	M	69	83	Heterozygous, 310–350 GAA repeats		Suspected TIA	+	+
5	M	74	76	Heterozygous, 510–520 GAA repeats	(+) (Mother)	Suspected vertebrobasilar ischemia	+	+
6	F	62	73	Heterozygous, 300–310 GAA repeats	+ (Mother)		+	+
7	F	75	93	Heterozygous, 290–295 GAA repeats	+ (Daughter)		+	+
8	F	73	83	Heterozygous, 255–265 GAA repeats		Suspected TIA	+	+
9	F	71	78	Heterozygous, 210–215 GAA repeats	(+) (Father)		+	+
10	F	74	79	Heterozygous, 310–320 GAA repeats	(+) (Mother)	Suspected pontine minor stroke; suspected TIA	+	+
11	F	63	68	Heterozygous, 425–445 GAA repeats in *FGF14*; additional heterozygous pathogenic AAGGG repeat expansion in *RFC1* of 345–365 repeats			-	
12	M	71	78	Heterozygous, 275–290 GAA repeats			+	-
13	F	79	94	Heterozygous, 225–235 GAA repeats in *FGF14*; additional expansion in *ATXN8/ATXN8OS* (SCA8), 63 ± 1 repeats on allele 1			-	
14	M	50	75	Heterozygous, 216–223 GAA repeats			-	
15	M	59	64	Heterozygous, 257–277 GAA repeats	(+) (Mother)	Two episodes of suspected TIA; one episode of suspected minor stroke	+	+
16	M	64	74	Heterozygous, 375–390 GAA repeats in *FGF14*; additional repeat expansions 150–155 in *ATXN8/ATXN8OS* (SCA8) Gen	-	Three episodes of suspected TIA	-	
17	M	37	67	Heterozygous, 320–360 GAA repeats in *FGF14*; with 210–230 repeats on the second allele of *FGF14* gene	(+) (Aunt)		+	+
18	W	52	78	Heterozygous, 420–425 GAA repeats	+ (Brother)	One Episode of suspected TIA	-	

*F* Female, *M* Male, *+* positive, *(+)* family history of gait disturbance without an established diagnosis, *TIA* transient ischemic attack

### Self-reported Clinical Symptoms

The most frequent initial manifestation was gait disturbance in 89% (16/18) of patients, followed by impaired balance in 56% (10/18). Vertigo was described by 17% (3/18) as the first presenting symptom.

During the disease course, gait disturbance was reported by all patients. Dysarthria was present in 94% of patients (17/18), fine-motor impairment in 61% (11/18), and diplopia in 61% (11/18). Other motor and non-motor symptoms were reported less frequently and are described in detail in Table 2.

**Table 2:** Neurological, Oculomotor, Episodic, and other Movement-Disorder Features in SCA27B

Clinical feature	*n*/18 (%)
Self-Reported Symptoms	
Gait disturbance	18/18 (100%)
Balance impairment	13/18 (72%)
Dysarthria	17/18 (94%)
Fine motor impairment	11/18 (61%)
Diplopia	11/18 (61%)
Vertigo/nausea	6/18 (33%)
Bulbar and respiratory features	
Dysphagia	7/18 (39%)
Chronic cough	5/18 (28%)
Non-motor Features & autonomic dysfunction	
Sleep disturbance	7/18 (39%)
Autonomic symptoms (any)	8/18 (44%)
Urinary dysfunction	6/18 (33%)
Orthostatic hypotension	4/18 (22%)
Oculomotor findings	
Slowed and hypometric saccades	18/18 (100%)
Broken-up smooth pursuit	13/18 (72%)
Square-wave jerks	2/18 (11%)
Downbeat nystagmus with gaze-evoked nystagmus	7/18 (39%)
Gaze-evoked nystagmus only	1/18 (5%)
Pathological vestibulo-ocular reflex (VOR)	13/18 (72%)
Expanded movement-disorder features	
Dystonia (any)	4/18 (22%)
Cervical dystonia	2/18 (11%)
Upper-limb dystonia	3/18 (17%)
Tremor	4/18 (22%)
Myoclonus	1/18 (5%)
Myokymia	1/18 (5%)
Parkinsonism	4/18 (22%)
Episodic course and triggers	
Episodic or fluctuating symptoms	16/18 (89%)
Triggered by stress	10/18 (56%)
Triggered by alcohol	4/18 (22%)
Triggered by caffeine	2/18 (11%)
Triggered by physical activity	2/18 (11%)
Triggered by positive emotional arousal	3/18 (17%)

Episodic symptoms or episodic worsening of symptoms were reported in 89%. In 27%, symptoms were initially episodic and later transitioned to a persistent course. The most frequently reported trigger factors were stress in 56% (10/18), alcohol in 22% (4/18), and caffeine in 11% (2/18); positive emotional arousal and physical activity were reported as triggers in 17% (3/18) and 11% (2/18) of patients, respectively. Furthermore, aggravation of the symptoms in the morning was described by 11% of the patients (2/18).

### Cognitive Function

MoCA and CCAS were available in sixteen patients. The mean MoCA score among all these patients was 25 [range 21 to 29]. Using the threshold of < 26 as pathological, 44% of patients (7/16) showed evidence of cognitive impairment on this scale.

The CCAS screening showed a mean total score of 86.9/120 (range 62–108). Based on the number of failed test items (1 = possible CCAS; 2 = probable CCAS; ≥3 = definite CCAS), 62% patients (10/16) met the criteria for definite CCAS, 19% (3/16) for probable CCAS, and 6% (1/16) for possible CCAS, whereas only 12% (2/16) performed within the normal range.

Correlation analysis of MoCA and CCAS revealed a weak-to-moderate positive association (Spearman ρ = 0.46), indicating that lower MoCA scores tended to be associated with lower CCAS total scores. However, this relationship was not statistically significant (*p* = 0.072), and several patients with normal MoCA scores showed clear CCAS abnormalities (Table 3).

**Table 3:** Neurological Examination and Paraclinical Findings

Patient No.	SARA	MoCA	CCAS (x/120; *n*/10)	Ataxic gait	Dysarthria	Myoclonus	Tremor	Dystonia	Pathological VOR	Slowed/hypometric saccades	Broken up smooth pursuit	SWJ	DBN	GEN	Cardio-vascular Risk	MRI	EMG	DAT-SPECT
**1**	13	24	80; 4	+	+	-	-	-	+	+	+	-	-	-	-	Parietal and cerebellar atrophy	NA	Nl
**2**	5	21	83; 3	+	+	+ (upper & lower limbs)	Intention Tremor	Cervical + upper limbs	-	+	+	-	-	-	-	Cerebellar atrophy	Bursts < 50 ms – myoclonus of the upper and lower limbs	NA
**3**	7	26	108; 0	+	+	-	-	-	-	+	-	-	-	-	HTN	Normal Findings		NA
**4**	10	26	91; 2	+	+	-	-	-	+	+	+	-	+	+	-	Cerebellar atrophy		NA
**5**	24,5	22	76; 5	+	+	facial myokymia	-	Upper limbs	+	+	+	-	+	+	HTN	Non-specific gliosis, no atrophy	Regular 5-Hz bursts (100 ms) in the right *levator alaeque nasi* muscle	NA
**6**	6	26	91; 2	+	+	-	-	-	+	+	-	-	+	+	-	Mild generalized brain volume reduction without cerebellar atrophy	NA	NA
**7**	12,5	26	62; 7	+	+	-	-	-	-	+	+	-	-	-	HTN	Microangiopathy, no atrophy	NA	NA
**8**	19	27	82; 4	+	+	-	Intention Tremor	Upper limbs	+	+	+	-	-	-	-	Mild bilateral white-matter demyelination; right supraventricular lacunar lesion	NA	Nl
**9**	11	25	73; 5	+	+	-	-	-	+	+	+	-	-	-	-	Global cerebral atrophy	NA	NA
**10**	7,5	27	84; 3	+	+	-	Postural Tremor	-	+	+	+	+	-	-	-	Predominantly central brain volume reduction; microangiopathy	NA	NA
**11**	5,5	25	101; 4	+	+	-	-	Cervical		+	-	-	-	-	-	Normal findings	NA	NA
**12**	11,5	26	103; 0	+	+	-	-	-	-	+	+	+	-	-	AF	Normal findings	NA	Nigrostriatal degeneration
**13**	16	25	87; 3	+	+	-	Postural Tremor	-	+	+	+	-	-	+	-	Moderate microangiopathic changes with cerebral volume loss not exceeding the age-expected range	Length-dependent chronic neurogenic changes of the lower limbs without active denervation -	NA
**14**	7	29	97; 1	+	-	-	-	-	+	+	+	-	+	+	-	Advanced supratentorial and infratentorial cerebral microangiopathy with focal cerebellar atrophy and no brainstem involvement	NA	NA
**15**	10	21	76; 7	+	+	-	-	-	+	+	-	-	+	+	DM	Cerebral microangiopathy; cerebellum preserved	NA	Nl
**16**	9	26	97; 2	+	+	-	-	-	+	+	+	-	+	+	HTN	NA	NA	NA
**17**	3,5	NA	NA	+	-	-	-	-	+	+	+	-	+	+	HTN	Normal Findings	Normal Findings	NA
**18**	8	NA	NA	+	+	-	-	-	+	+	+	-	-	-	-	Post traumatic brain injury with large right temporal parenchymal defect, cerebral microangiopathy	NA	NA

*SARA* = Scale for the Assessment and Rating of Ataxia, *MoCA *= Montreal Cognitive Assessment, *CCAS *= Cerebellar Cognitive Affective Syndrome (1 = possible CCAS, *2* = probable CCAS, *≥3* = definite CCAS), *VOR *= Vestibulo-Ocular Reflex, *SWJ *= Square Wave Jerks, *DBN *= Downbeat Nystagmus, *GEN *= Gaze Evoked Nystagmus, *MRI *= magnetic resonance imaging, *EMG *= electromyography, *DAT-SPECT *= dopamine transporter single-photon emission computed tomography, *NA* = Not available, *Nl* = Normal findings, *HTN* = Hypertension, *AF *= Atrial Fibrillation, *DM *= Diabetes Mellitus

There was a moderate negative correlation between SARA and MoCA scores (Spearman ρ = −0.57; *p* = 0.02). Furthermore, there was a negative association between SARA and CCAS total scores (Spearman ρ = −0.66, *p-value* < 0.01), indicating a poorer CCAS and MoCA performance with increasing ataxia severity.

Vascular risk factors were present in 6 of 18 patients (33.3%), including arterial hypertension (*n* = 5), and diabetes mellitus (*n* = 1). Chronic microangiopathic MRI changes were observed in 6 of 17 patients (35%). Among patients with available cognitive assessments (*n* = 16), cognitive abnormalities were observed both in individuals with and without cardiovascular risk factors or microangiopathic MRI changes.

### Neurological Examination Findings

All patients showed a cerebellar syndrome on standardized neurological examination. Gait disturbance was present in all patients (18/18). Dysarthria was observed in 89% (16/18) and dysdiadochokinesia in 56% (10/18). The severity of cerebellar impairment, as assessed by the mean SARA, was 10.1 points [ranged 3.5 to 24.5].

Most patients exhibited a distinctive gait pattern. During straight-line walking, patients repeatedly deviated laterally from the walking line, performing small corrective sidesteps to either side rather than demonstrating a broad-based staggering gait or a tendency to fall. Patients did not report a subjective falling sensation, and falls were not observed, despite the recurrent side-to-side veering while walking.

This “lateral veering gait with corrective sidesteps” was a gait pattern that was observed consistently across several patients in this study (Video1).

Oculomotor abnormalities were frequently observed. Slowed and hypometric saccades were present in all patients (18/18), broken smooth pursuit in 72% (13/18), and square-wave jerks in 11% (2/18). Downbeat nystagmus on fixation, together with gaze-evoked nystagmus, was observed in 39% (7/18), and pathological vestibulo-ocular reflex (VOR) was identified in 72% (13/18).

Dystonia was seen in four patients. One patient had segmental dystonia, with cervical and upper extremities involvement, a second patient presented with cervical dystonia, and two further patients exhibited focal dystonia of the upper extremities without cervical involvement.

Moreover, four patients presented with tremor of the upper limbs (two with intention tremor, two with postural tremor).

One patient had irregular, stimulus-sensitive myoclonus of the upper and lower limbs, confirmed neurophysiologically by surface electromyography (EMG), which demonstrated short burst durations < 50 ms in the arms and legs (Table 3, Patient 2). In another patient, involuntary movements of the face, which EMG characterized as regular 5-Hz bursts of approximately 100 ms in the musculus levator alaeque nasi on the right side, were consistent with myokymia (Table 3, Patient 5) (Video 2).

Parkinsonism was observed in four patients, consisting of combinations of bradykinesia and rigidity on clinical examination, occurring in the absence of resting tremor. In one of these patients, dopamine transporter imaging (DATSCAN) showed nigrostriatal degeneration, whereas the remaining patients showed parkinsonism in the neurological examination without pathological DATSCAN.

### Treatment Response to 4-aminopyridine

A total of 12 patients received treatment with 4-aminopyridine (10 mg twice daily). Among these, 83% reported an improvement of their neurological symptoms, whereas two patients did not report a relevant benefit.

The most frequently reported improvements were gait stability and walking endurance, followed by improvement of dysarthria, balance, and fine motor function. Several patients additionally described a reduction in the frequency and severity of episodic symptom exacerbations. Two patients reported that episodic nausea and vomiting as part of their symptom spectrum were markedly improved after treatment initiation.

No serious adverse events were observed. Treatment was generally well-tolerated, and no patient discontinued therapy due to side effects.

### Additional Investigations

Brain MRI was available for 17 patients. Detailed MRI findings are demonstrated in Table 3. Cerebellar atrophy was present in 4 of 17 patients, in some cases with additional supratentorial volume loss. In 7 of 17 patients, the predominant finding consisted of supratentorial microangiopathy without relevant cerebellar atrophy, whereas 5 patients had essentially unremarkable MRI findings.

Neurophysiological studies were available for 9 patients, that in 7 of them showed evidence of peripheral neuropathy, most commonly with a predominant axonal, sensory, or sensorimotor pattern.

### Previous Diagnoses

Diagnostic misclassification was frequent in the early disease stages, particularly in patients who initially presented with episodic symptoms. In 39% of patients, the recurrent transient episodes had previously been interpreted as transient ischemic attacks (TIA), and these individuals received cerebrovascular work-up and treatment before the later development of a persistent cerebellar syndrome. In another patient, recurrent stereotypic episodes of isolated dysarthria were thought to be epileptic seizures, despite multiple normal EEG examinations. One patient had previously been misdiagnosed with multiple system atrophy (MSA) because of the syndrome consisting of gait disturbance, autonomic symptoms, parkinsonism and cerebellar signs.

## Discussion

In this study, we provide a comprehensive clinical characterization of a patient cohort with SCA27B to expand the currently known phenotypic and genetic spectrum of this disease.

### Genetic Findings

In our cohort of adult-onset ataxia, 24% of patients tested positive for SCA27B. Among the SCA27B patients, 83% carried a pathogenic *FGF14* GAA repeat expansion of more than 300 repeats or within the range of 250–300 repeats. Notably, 17% of patients exhibited lower-range expansions of 200–250 repeats, who nonetheless also displayed the typical clinical phenotype of SCA27B, with no apparent differences in symptom profile or disease severity. These findings further support recent observations suggesting the pathological threshold for *FGF14* GAA repeat expansions may be lower than previously assumed [4].

Another important finding of the present study is the identification of dual heterozygous gene repeat expansions in three patients: the co-occurrence of a pathogenic *FGF14* GAA repeat expansion with a heterozygous pathological *RFC1* expansion in one patient and a heterozygous *ATXN8/ATXN8OS* (SCA8) repeat expansions in two patients. The first report of two siblings with biallelic pathogenic *RFC1* and heterozygous *FGF14* expansions was recently published by Tsokkos et al., presenting with progressive gait ataxia, vestibular dysfunction, and sensory neuronopathy, one of whom exhibited episodic symptoms [19]. Another sibling carried a heterozygous pathogenic *RFC1* and a *FGF14* expansion presenting with a pure SCA27B phenotype [19]. Our patient, therefore, represents the second reported case of a dual repeat expansion involving pathogenic heterozygous *FGF14* and *RFC1* expansions. As the carrier frequency of a heterozygous *RFC1* expansion is 1 in 14 [20], the dual repeat expansions in our patient are most likely a coincidence.

Moreover, the co-occurrence of *FGF14* and *ATXN8/ATXN8OS* repeat expansions represents, to our knowledge, the first report of this genetic combination. SCA8 is known for its highly variable penetrance and phenotypic expressivity, ranging from asymptomatic carriers to individuals with progressive cerebellar ataxia and multisystem involvement [21]. Both *FGF14* and *ATXN8/ATXN8OS* are located on chromosome 13, at chromosomal regions 13q33.1[3] and 13q21 [22], respectively. These findings contribute to the emerging recognition that adult-onset cerebellar ataxias may, in a subset of patients, result from combined repeat expansion disorders, rather than a single monogenic etiology, and further support the concept that these repeat expansions may coexist and contribute jointly to complex and overlapping phenotypes. This has important implications for diagnostic strategies, genetic counseling, and the interpretation of genotype–phenotype correlations in SCA27B and related ataxia syndromes.

### Clinical and Neurological Examination Findings

The clinical spectrum of SCA27B observed in our cohort was mostly consistent with previously reported phenotypes. Gait disturbance (100%), dysarthria (89%), and dysdiadochokinesia (56%) represented the most frequent neurological findings in our patient, in line with prior studies [23]. As described in earlier reports, an episodic disease course was a core clinical feature of SCA27B in our patients. Previous studies have reported episodic symptoms in approximately 28–72% of cases, often triggered by alcohol intake or physical exertion [8, 23, 24]. In our cohort, episodic manifestations were observed in 89% of patients. The most reported trigger factors were stress (56%), alcohol (22%), and positive emotional arousal (17%), followed by caffeine consumption (11%) and physical activity (11%). The slightly higher prevalence of episodic symptoms in our study is likely attributable to the limited sample size and the systematic phenotyping approach applied.

Oculomotor abnormalities likewise represented a prominent component of the SCA27B phenotype, comparable to other forms of spinocerebellar ataxias (SCA). Cerebellar ocular motor signs, including hypometric saccades (100%), downbeat nystagmus (39%), pathological VOR (72%), and gaze-evoked nystagmus (44%), were among the most frequently observed abnormalities in our cohort, consistent with previously published findings [25–27]. These findings may provide useful diagnostic clues in patients with episodic or atypical presentations.

We also observed a typical gait pattern in our cohort characterized by recurrent lateral veering with corrective sidesteps during forward walking, in the absence of falling tendency or marked postural instability. This gait phenotype differs from the broad-based, ataxic gait commonly described in spinocerebellar ataxias, as well as from the gait impairment associated with bilateral vestibulopathy, such as in CANVAS or toxic vestibulopathy, in which gait disturbance is usually characterized as broad-based with a pronounced tendency to fall [28, 29]. Hence, this gait disturbance may represent a recognizable clinical hallmark of SCA27B. Future studies incorporating quantitative gait analysis may help to further describe this pattern precisely and assess its specificity.

A central and novel finding of the present study was the identification of additional movement-disorder manifestations beyond the classical cerebellar syndrome.

22% of our cohort presented with isolated or segmental dystonia. Although dystonia is a known reported feature in several spinocerebellar ataxia subtypes, including SCA1, SCA2, SCA3, SCA6, SCA14, and SCA21 [30–35], it has only been reported in one recent study in SCA27B patients [36].

Moreover, tremor has previously been reported in approximately 22% of patients with SCA27B [24]. In our cohort, also 22% of patients exhibited upper limb tremor, consistent with earlier observations. Given its close association with cerebellar dysfunction, tremor is nevertheless most probably a manifestation of the cerebellar syndrome rather than being a distinct movement disorder feature.

Furthermore, one patient presented with electromyographically confirmed myoclonus of the upper and lower limbs, and another patient presented with facial myokymia. To our knowledge, this is the second report of myokymia [24] and the first report of myoclonus in SCA27B. Myoclonus in spinocerebellar ataxias is thought to be due to dysfunction of the cerebellar–thalamo–cortical pathway, leading to a loss of cerebellar inhibitory control over the motor cortex and, consequently, increased cortical excitability [37, 38]. Clinically, myoclonus is most frequently observed in SCA14 and SCA1 [35, 37]. Myokymia, on the other hand, is commonly linked to peripheral nerve hyperexcitability [39]. In the present cohort, neurophysiological confirmation of myoclonus and myokymia raises the possibility that the *FGF14* repeat expansion may lead to broader network dysfunction affecting cerebellar–cortical motor loops.

Noteworthy is the observation of parkinsonism with a combination of both rigidity and bradykinesia in 27% of the patients in our cohort, with one individual demonstrating dopaminergic degeneration on DaTSCAN imaging. Previous reports have described parkinsonism in SCA27B only sporadically, mostly as an incidental finding that may be explained by alternative etiologies [4, 40, 41]. Parkinsonism has nevertheless been reported in several SCA subtypes, most notably SCA2, SCA3, and SCA17, and less frequently in SCA6 and SCA8 [42, 43]. Hence, our findings suggest that parkinsonian features may represent an additional and previously underrecognized component of the SCA27B motor phenotype, and support the concept of SCA27B is a disorder affecting not only cerebellar but also basal ganglia motor circuits.

Treatment with 4-aminopyridine resulted in subjective and clinical improvement in 82% of treated patients in our cohort, consistent with previous reports [44, 45].

### Cognitive Function

A further major finding of the present study is the high prevalence of cognitive impairment among patients with SCA27B. Cognitive impairment in the form of CCAS has been described to be more prevalent in SCA27B compared to other SCAs [11]. Two-thirds of patients in our cohort fulfilled the criteria for definite CCAS, indicating impairment across multiple cognitive domains typically associated with cerebellar dysfunction. Additionally, a substantial proportion of patients showed reduced performance on MoCA, further supporting the presence of mild to moderate cognitive impairment.

The cognitive profile observed in our cohort is consistent with the concept of the cerebellum as a key structure within distributed cognitive networks [11]. Hence, the high frequency of CCAS in SCA27B suggests that *FGF14* repeat expansions may disrupt cerebello–cortical circuits involved in higher-order cognitive processing, extending the clinical phenotype beyond a purely motor cerebellar syndrome. Notably, a substantial subset of our patients fulfilled the criteria for definite CCAS despite exhibiting normal performance on the MoCA test, indicating that CCAS screening may be more sensitive for detecting cerebellar-related cognitive dysfunction in this population. Furthermore, our results showed a worse CCAS and MoCA performance with increasing ataxia severity as assessed by the SARA score. These findings have direct clinical implications, as cognitive symptoms contribute to disease burden and functional impairment but may remain underrecognized in routine neurological practice.

### Previous Diagnoses

An important finding of our study, relevant in clinical practice, is the risk of diagnostic misclassification in patients with SCA27B, particularly during the early and episodic stages of the disease. In our cohort, 39% of the patients had previously been diagnosed with transient ischemic attacks (TIA) because of recurrent episodic cerebellar symptoms, and one patient with epilepsy due to recurrent monomorphic dysarthria. In addition, one patient had initially been diagnosed with multiple system atrophy.

The episodic nature of SCA27B, may be particularly misleading in the early disease phase, when persistent cerebellar signs are subtle or absent. In such cases, transient neurological deficits are frequently interpreted as cerebrovascular events, especially in older patients with vascular risk factors. Similarly, the presence of autonomic and parkinsonian features may raise suspicion of MSA, further complicating the diagnostic process.

Our findings emphasize the importance of considering SCA27B in the differential diagnosis of adult-onset episodic or fluctuating cerebellar syndromes, particularly when neuroimaging is unrevealing, and repetitive symptoms emerge. Early recognition is clinically relevant, as symptomatic treatment with 4-aminopyridine may improve symptoms, and accurate diagnosis allows for appropriate genetic counseling.

### Limitations

Several limitations of the present study should be acknowledged. First, the cohort size is relatively small, reflecting the single-center design. Hence, prevalence estimates of individual clinical features should be interpreted with caution and require confirmation in larger multicenter cohorts. Second, the cross-sectional design limits conclusions regarding the longitudinal evolution of motor, cognitive, and non-motor symptoms. Longitudinal follow-up studies are necessary to further understand the dynamics of symptoms. The third limitation relates to the interpretation of *FGF14* repeat expansions in the range of 200–250 repeats in three of the patients. Although recent studies have proposed that expansions above 200 repeats may be pathogenic in the presence of a compatible phenotype, the current pathogenic threshold is still defined by > 250 repeats [4]. Therefore, the possibility of alternative causes responsible for the ataxic phenotype in this number of patients was not completely excluded, as no additional exome or genome sequencing and segregation analyses were done. Nevertheless, repeat expansion disorders represent a significantly more common genetic cause of adult-onset ataxia than other causes detected by exome sequencing [14], which makes missed alternative genetic diagnoses less likely, particularly considering the fact that these three patients all presented with a phenotype highly consistent with the known SCA27B phenotype. Another important limitation of this study is the absence of an age- and education-matched control group for the cognitive assessment. Hence, cognitive abnormalities cannot be exclusively linked to SCA27B. Although cardiovascular risk factors and chronic microangiopathic MRI changes did not show an obvious relationship with cognitive performance in our cohort, the role of age-related or vascular factors cannot be completely excluded due the small sample size. Future controlled studies are needed to better define the cognitive profile of SCA27B and to distinguish the disease-specific cerebellar cognitive dysfunction from effects related to aging, vascular risks, and educational level.

## Conclusion

In conclusion, this study provides a comprehensive clinical presentation of patients with SCA27B and expands the currently recognized phenotypic spectrum of this disorder. In addition to confirming the core cerebellar phenotype with episodic and progressive gait ataxia and prominent oculomotor dysfunction, we identified myokymia, myoclonus, dystonia, and parkinsonism as previously less recognized manifestations of SCA27B. Furthermore, we show that cognitive impairment and CCAS are frequent and clinically relevant findings in SCA27B, although further studies are required to demonstrate that this is a specific component of the disease.

The high rate of diagnostic misclassification observed in our cohort highlights the importance of considering SCA27B in the differential diagnosis of adult-onset episodic or fluctuating cerebellar syndromes.

Together, these results contribute to a more comprehensive understanding of SCA27B and provide a framework for future multicenter studies aimed at refining the genotype–phenotype correlation.

## Supplementary Information

Below is the link to the electronic supplementary material.

Supplementary Material 1 (DOCX 16.1 KB)

Supplementary Material 2 (MOV 20.6 MB)

Supplementary Material 3 (MP4 36.0 MB)

## Data Availability

No datasets were generated or analysed during the current study.
