# Supplementary material for: Phenotype and Genetics of Spinocerebellar Ataxia Type 27B: Novel Movement-disorder Features, Cognitive Impairment, and Repeat Expansion Findings
Source: Cerebellum. 2026 Jun 29;25(4):102. doi: 10.1007/s12311-026-02041-y (PMC13315097; doi:10.1007/s12311-026-02041-y)
Supplement: Supplementary file 1 — Supplementary Material 1 (DOCX 16.1 KB) [file 12311_2026_2041_MOESM1_ESM.docx]

Supplementary Material 1. Summary of established diagnoses among patients with late-onset Ataxia

| **Diagnosis** | **Number of identified patients** |
| --- | --- |
| Without an established Diagnosis | 39 |
| Not tested for SCA27B | 25 |
| Tested negative for SCA27B | 14 |
| SCA27B | 18 (additionally 1 patient with 175-210 repeat expansions and 1 patient 180-185 repeat expansions) |
| Cerebellar Ataxia, Neuropathy, and Vestibular Areflexia Syndrome (CANVAS-Syndrome) | 6 |
| Alpha-methylacyl-CoA racemase (AMACR) deficiency | 1 |
| SCA1 | 4 |
| SCA2 | 3 |
| SCA3 | 3 |
| SCA4 | 1 |
| SCA6 | 1 |
| SCA 8 | 1 |
| SCA10 | 1 |
| SCA28 | 1 |
| SCA36 | 2 |
| SCA47 | 1 |
| MSA-C | 4 |
| Ataxia with Oculomotor Apraxia Type 2 | 4 |
| Spinocerebellar Ataxia with Axonal Neuropathy 3 (SCAN3) | 1 |
| Progressive Ataxia and Palatal Tremor (PAPT) | 1 |
| Hereditary Spastic Paraplegia Type 7 (SPG7) | 1 |
| Kearns-Sayre Syndrome | 1 |
| Late onset Friedreich's ataxia | 1 |
| Autosomal recessive spastic ataxia type 5 (SPAX5) | 1 |
| Cerebellar syndrome and spastic paraparesis associated with a pathogenic variant in *MT-WD4* | 1 |
